# Enhancement of Anti-Hypoxic Activity and Differentiation of Cardiac Stem Cells by Supernatant Fluids from Cultured Macrophages that Phagocytized Dead Mesenchymal Stem Cells

**DOI:** 10.3390/ijms17071175

**Published:** 2016-07-20

**Authors:** Liang Liu, Xian Jin, Zhong’e Zhou, Chengxing Shen

**Affiliations:** 1 Department of Cardiology, Xinhua Hospital, Shanghai Jiao Tong University School of Medicine, 1665 Kongjiang Road, Shanghai 200092, China; liuliangsuda@163.com; 2 Department of Cardiology, Central Hospital of Minhang District, 170 Xinsong Road, Shanghai 201199, China; jinxianian@126.com (X.J.); zhouzhonge2006@126.com (Z.Z.)

**Keywords:** macrophages, dMSC, CSCs, growth factors, differentiation

## Abstract

**Background:** Most mesenchymal stem cells (MSCs) die shortly after transplantation into a myocardial infarcted area. Dead MSCs (dMSCs) are phagocytized by macrophages (pMΦ) in vivo and in vitro; however, the effects of pMΦ on cardiac stem cells (CSCs) remain unknown. **Methods:** MSCs, CSCs, and macrophages were obtained from bone marrow, hearts, and peritoneal cavity of mice, respectively. dMSCs were harvested after hypoxia for 24 h, and incubated with macrophages (2:1) for another 2 days with or without lipopolysaccharide (LPS, 50 ng/mL) and sorted by flow cytometry to obtain pMΦ. Viability and apoptosis of CSCs were respectively evaluated with the cell counting kit-8 (CCk-8) assay and Annexin V-PE/7-AAD staining at 0, 6, 12, and 24 h of culture with supernatant fluids from macrophages (MΦ), LPS-stimulated macrophages (LPS-pMΦ), pMΦ, and MSCs. GATA-4 and c-TnI expression was measured by flow cytometry on the seventh day. Expression of inflammation and growth factors was assessed by real-time polymerase chain reaction (RT-PCR) in MΦ, LPS-pMΦ, and pMΦ cells. **Results:** pMΦ expressed higher levels of interleukin-10 (IL-10) and transforming growth factor-β (TGF-β)and lower levels of tumor necrosis factor-α(TNF-α)and IL-6 than LPS-pMΦ, higher levels of growth factors and of GATA-4 and c-TnI at the 7th day, which were similar to those in MSCs. CSCs cultured with supernatant fluids of pMΦ exhibited higher proliferative, anti-hypoxic, and differentiation activities. **Conclusion:** The supernatant fluids of macrophages that had phagocytized dead MSCs encouraged changes in phenotype and growth factor expression, enhanced proliferation, differentiation, and anti-hypoxic activity of CSCs, which is relevant to understanding the persistent therapeutic effect of MSCs after their massive demise upon transplantation in myocardial infarction. Furthermore, some miRNAs or proteins which were extracted from the supernatant fluids may give us a new insight into the treatment of myocardial infarction in the future.

## 1. Introduction

Cardiovascular disease, especially myocardial infarction, remains the leading cause of mortality and morbidity around the world [1] and the focus of intense clinical and basic research—in particular on transplantation of stem and progenitor cells to rescue the injured myocardium [2,3].

Bone marrow-derived mesenchymal stem cells (MSCs), a type of multipotent stem cell that can differentiate into various cell types, such as osteoblasts, muscle cells, and cardiomyocytes [4,5], have unique immunological characteristics that minimize immune rejection [5,6] and render them most suitable for stem cell therapy, as evidenced by basic science and clinical studies on their benefits for myocardial healing [7,8]. However, the hypoxic–ischemic and inflammatory microenvironments in the ischemic myocardium lowers survival and viability of MSCs [9]. In a previous study, we demonstrated that MSCs transplanted into a porcine myocardial ischemia/reperfusion model were engulfed by cardiac macrophages, which then had a beneficial effect on cardiac function [10]. Moreover, our in vitro study showed that supernatant fluids obtained from macrophages that phagocytized dead MSCs (pMΦ) improved survival of hypoxic cardiomyocytes [11]. Although the main mechanisms underlying the therapeutic effects of MSCs remain unknown, pMΦ may thus play an important role.

Recent studies have documented the presence of a small amount of cardiac stem cells (CSCs) in the postnatal heart, which have been regarded as one of the most promising stem cell types for myocardial protection and restoration [12,13]. The therapeutic effects of CSCs are considered secondary to paracrine mechanisms and cardiovascular lineage differentiation [14,15]. However, it remains undefined how pMΦ affect CSCs. Therefore, in the present study, our hypothesis was that whether the supernatant fluids obtained from pMΦ could induce CSC differentiation into cardiomyocytes and enhanceanti-hypoxic and proliferative activities. We showed that lipopolysaccharide (LPS)-stimulated macrophages can phagocytize dead MSCs and express more growth and anti-inflammatory factors that are similar to MSCs. Exposure to the supernatants of pMΦ enhanced the anti-hypoxia, proliferation, and differentiation properties of CSCs. In the future, some miRNAs or proteins can be extracted from the supernatant fluids that could directly act as a therapy for myocardial infarction. These results give us a new insight into the treatment of myocardial infarction.

## 2. Results

### 2.1. Lipopolysaccharide (LPS)-Stimulated Macrophages Phagocytosed Dead Mesenchymal Stem Cells (dMSCs)

**Figure 1:**
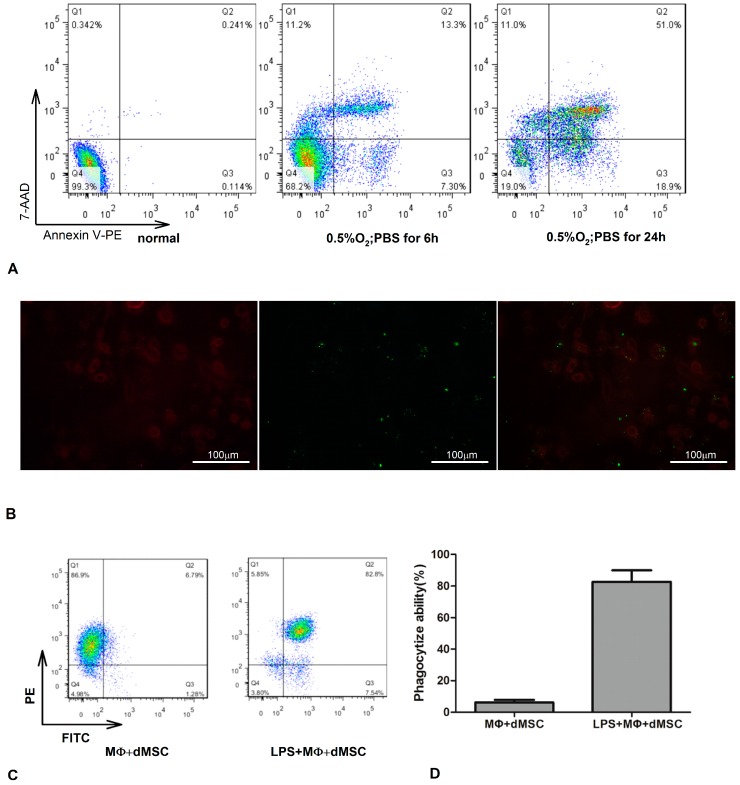
Dead mesenchymal stem cells (dMSCs) were phagocytosed by lipopolysaccharide (LPS)-stimulated macrophages. dMSC labeled with FITC-green fluorescent protein (GFP) and activated macrophages (2:1) stained with PE-F4/80 were co-cultured for 48 h, and then detected by fluorescence microscopy and flow cytometry (FCM). (**A**) MSCs were cultured with three conditions, including no hypoxia and 10% fetal bovine serum (FBS), 0.5% O_2_ for 6 h and phosphate buffered saline (PBS), 0.5% O_2_ for 24 h and PBS. The ratio of dMSCs was the greatest in third group; (**B**) Fluorescence microscopy was used to show the phenomenon of phagocytosis; (**C**,**D**) Phagocytosis ability of macrophage was evaluated by FCM. Significant difference was shown in two groups with or without LPS (*p* < 0.001), and the mean phagocytosis rate (%) of LPS-stimulated macrophage was 80%. The percentage of each group and each bar represents the mean value of triplicate results, which means three independent experiments were done. MΦ: macrophage.

In our previous research, we found that macrophages stimulated by LPS exhibited enhanced phagocytosis of dMSCs [11]. To verify this phenomenon in the present study, dMSCs were measured by flow cytometry (FCM) in three groups. As shown in Figure 1A, the proportion of dMSCs was 0.7%, 31.8%, and 81% in the no hypoxia, 10% fetal bovine serum (FBS); 0.5% O_2_ for 6 h, phosphate buffered saline (PBS); and 0.5% O_2_ for 24 h, PBS groups, respectively. The optimalmultiplicity of infection (MOI) of green fluorescent protein (GFP) transfection was 10.The transfection rate was almost 90% (not provided). We then co-cultured with or without LPS (50 ng/mL)-stimulated macrophages labeled with PE-anti-F4/80 and dMSC (cultured by 0.5% O_2_ and PBS for 24 h) marked with the FITC-GFP for 48 h, at a cell ratio of 1:2. dMSCs were engulfed by LPS-stimulated macrophages, as was obviously visible under fluorescence microscopy (Figure 1B). The FITC-GFP and PE-anti-F4/80 double-stained cells almost reached 80% as detected by FCM, and then the double-stained cells were sorted by FCM and defined as “pMΦ” (Figure 1C,D).

### 2.2. Inflammatory Cytokines Released by Macrophages (MΦ), Lipopolysaccharide (LPS) + MΦ, Phagocytized by Macrophages (pMΦ)

**Figure 2:**
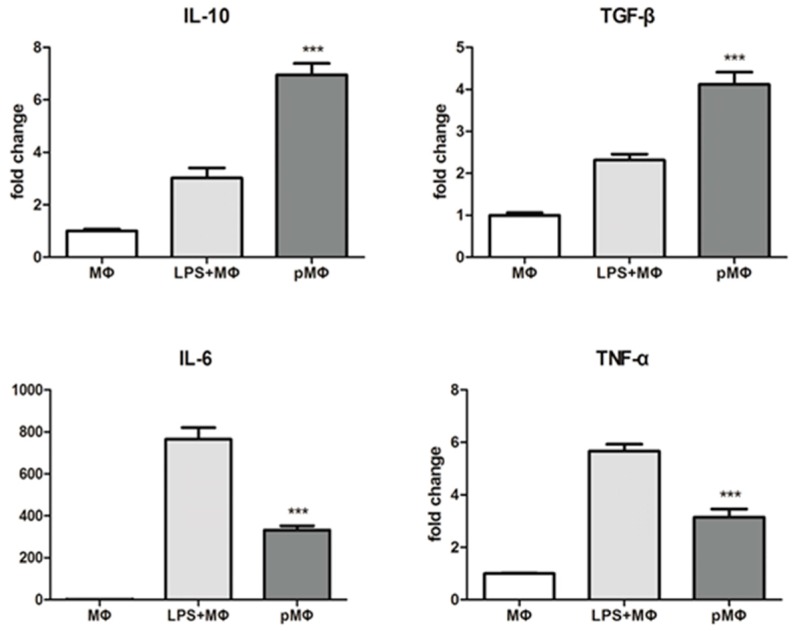
Inflammatory factors secreted by the cell of MΦ, LPS + MΦ, and pMΦ. Anti-Inflammatory factors interleukin-10 (IL-10) and transforming growth factor-β (TGF-β) had higher expression in pMΦ, and pro-Inflammatory factors IL-6 and tumor necrosis factor-α (TNF-α) had lower expression in pMΦ. *** *p* < 0.001 compared with MΦ. Each bar represents the mean value ± SD of triplicate results, which means three independent experiments were done.

Inflammatory cytokines secreted by each group were detected by real-time polymerase chain reaction (RT-PCR). Relative expression of the pro-inflammatory cytokines interleukin-6 (IL-6)and tumor necrosis factor-α (TNF-α) [16] in pMΦ was lower than in LPS + MΦ (*p* < 0.001), while that of the anti-inflammatory cytokines IL-10 and transforming growth factor-β (TGF-β) was higher in pMΦ than in LPS + MΦ (*p* < 0.001, Figure 2). Therefore, these findings show that pMΦ appeared to have adopted a more anti-inflammatory phenotype that may be beneficial to injured cells and tissues.

### 2.3. Growth Factors Released by pMΦ, MΦ and LPS + MΦ and Mesenchymal Stem Cells (MSCs)

**Figure 3:**
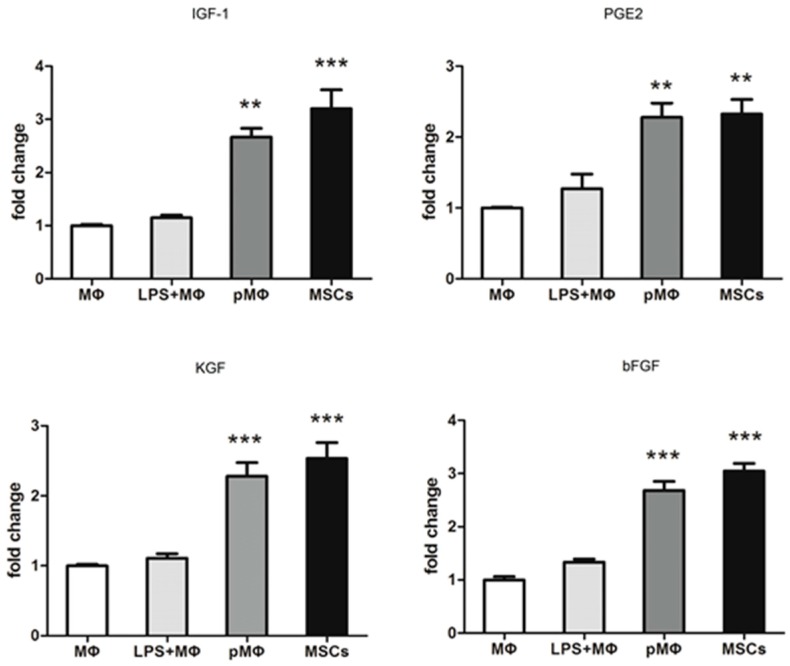
Growth factors were secreted by the cell of MΦ, LPS+ MΦ, pMΦ, and MSCs. Compared to MΦ, all growth factors (Insulin like Growth Factor-1 (IGF-1), Phenyl Glycidyl Ether2 (PGE2), Keratinocyte Growth Factor (KGF), bFibroblast Growth Factor (bFGF)) showed significantly higher expression in pMΦ, and the results were similar or approaching that of the MSCs (** *p* < 0.01, *** *p* < 0.001). Each bar represents the mean value ±SD of triplicate results, which means three independent experiments were done.

RT-PCR was utilized to measure the relative mRNA expression of growth factors in MΦ, LPS + MΦ, pMΦ, and MSCs group’s cells. Relative mRNA expression for insulin like growth factor-1 (IGF-1), phenyl glycidyl ether2 (PGE2), keratinocyte growth factor (KGF) and bfibroblast growth actor (bFGF) in pMΦ was significantly higher than in MΦ and LPS + MΦ, and similar or approaching that in MSCs (Figure 3).

### 2.4. Supernatant Fluids of pMΦ Improved Anti-Anoxia and Proliferation of Cardiac Stem Cells (CSCs)

**Figure 4:**
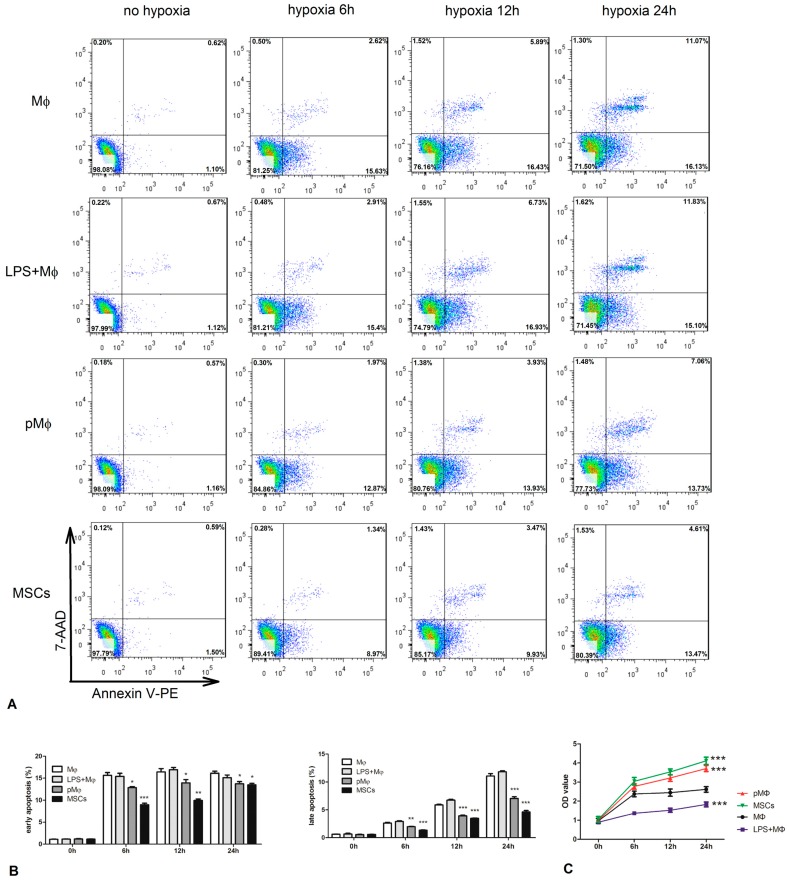
Cell counting kit-8 (CCK-8) proliferation assay and Annexin V-PE/7-ADD detected by FCM were used to measure the proliferation and anti-hypoxic properties of CSCs. (**A**,**B**) CSCs which were co-cultured with the supernatants of MΦ, LPS-MΦ, pMΦ, and MSCs were harvested in 0, 6, 12, and 24 h in hypoxia (0.5% O_2_) conditions. Compared to the MΦ and LPS-MΦ, the proportion of apoptosis in CSCs was significantly lower in pMΦ and MSCs at each time point; (**C**) The proliferation of CSCs detected by CCK-8 was dramatically increased in pMΦ and MSCs groups.* *p* < 0.05; ** *p* < 0.01; and *** *p* < 0.001 compared with MΦ. The percentage of each group represents the mean value of triplicate results, which means three independent experiments were done.

CSCs were cultured with supernatant fluids which were drawn from MΦ, LPS-MΦ, pMΦ, and MSCs, and then placed in an anoxic environment with a lack of nutrition (0.5% O_2_, PBS) for 0, 6, 12, and 24 h. At each time point, cells were collected and apoptosis was analyzed by FCM. The anti-anoxia status of CSCs was significantly enhanced at 6, 12, and 24 h by the presence of supernatant fluids from pMΦ (Figure 4A,B). CSCs proliferation assessed using cell counting kit-8 (CCK-8)—was increased in all groups (Figure 4C), but was significantly higher in pMΦ and MSCs than in MΦ at 6, 12, and 24 h.

### 2.5. Supernatant Fluids of pMΦ Induced CSCs Differentiation

**Figure 5:**
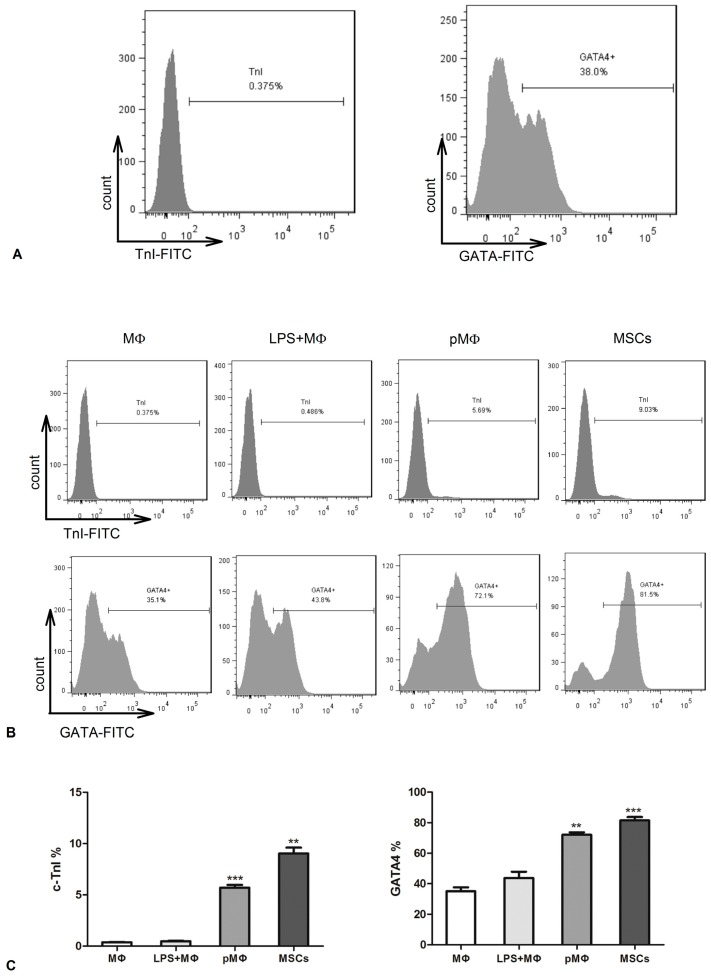
The markers of cardiomyocytes were detected in CSCs. (**A**) CSCs were obtained from the hearts of mice and then GATA-4 and TnI were detected by FCM; (**B**,**C**) CSCs co-cultured with the supernatants of MΦ, LPS-MΦ, pMΦ, and MSCs were harvested after 7 days. FCM was used to detect cardiomyocytes markers. GATA-4 and TnI showed significantly higher expression in the pMΦ and MSC groups; ** *p* < 0.01; and *** *p* < 0.001, compared with MΦ. The percentage and the bar of each group represent the mean value of triplicate results, which means that three independent experiments were done.

CSCs markers were identified by FCM. After isolation, CSCs had high expression of GATA-4 (38%) and low expression of TnI (0.375%, Figure 5A). The CSCs were cultured for 7 days in the presence of supernatant fluids from MΦ, LPS-MΦ, pMΦ, and MSCs. GATA-4 and TnI were significantly higher in pMΦ than MΦ and LPS-MΦ, with similar levels between pMΦ and MSCs (Figure 5B,C). Seven days later GATA-4 and TnI of CSCs in the presence of the supernatant fluids of pMΦ almost attained 5.69% and 72.1%, which was significantly higher than MΦ and LPS-MΦ, and similar to the levels of the MSC group (Figure 5B,C).

## 3. Discussion

In the present study, LPS-stimulated macrophages phagocytized dMSCs (pMΦ) and then expressed more growth and anti-inflammatory factors and less pro-inflammatory factors. CSCs cultured in the presence of the supernatant fluids of these pMΦ not only exhibited enhanced proliferative and anti-hypoxic activities, but also differentiation. To our knowledge, this study is the first to document the beneficial effects of pMΦ on CSCs, which might constitute one of the mechanisms underlying the persistence of therapeutic effects of dMSCs despite their massive demise after transplantation into myocardial infarction (MI) models.

Although the specific mechanisms whereby MSCs promote improved cardiac function has been undefined, it is known that most MSCs die shortly after implantation because of the unwelcoming microenvironments of MI [17,18,19], and that apoptotic cells are phagocytized by activated macrophages [20,21]. Phagocytosis of dMSCs by activated macrophages could mediate the observed effects of MSCs after their demise. In a previous study, MSCs that overexpressed the heme oxygenase-1 (HO-1) gene and stained with super paramagnetic iron oxide (SPIO) significantly improved cardiac function compared with other MSCs after transplantation into a porcine myocardial ischemia/reperfusion model. However, one week later, signal voids caused by the SPIO were observed by MRI in the surrounding area of myocardial infarction, reflecting that cardiac macrophages had phagocytized SPIO-labeled MSCs, as shown by histological analysis [10]. In our study, we found that activated macrophages induced by LPS phagocytized dMSCs, which was consistent with the aforementioned in vivo and in vitro studies [10,11]. We could thus infer that, compared to normal macrophages, pMΦ may have changed and played a similar function to MSCs on the repair of the injured heart.

Activated macrophages can be divided into M1 and M2 macrophages [16,22,23]. M1 phenotype macrophages (classically activated macrophages) mount inflammatory reactions by secreting many pro-inflammatory cytokines, such as IL-1β and TNF-α, among others [16], while M2 phenotype macrophages (alternatively activated macrophages) secrete many anti-inflammatory cytokines such as IL-10, Arg-1, and TGF-β, involved in anti-inflammatory reactions and tissue remodeling [16]. In the present study, we found that compared with LPS-MΦ, pMΦ exhibited upregulated IL-10 and TGF-β expression and downregulated IL-1 and TNF-α expression, which might reflect a pMΦ-induced macrophage polarization toward an M2 phenotype compatible with tissue repair, and with the observed enhanced anti-hypoxic and anti-apoptotic activities of CSCs similar to those of MSCs at different time points.

Another important finding of the present study was that expression of GATA-4 (72.1%) and c-TnI (5.69%) by CSCs as detected by FCM was significantly increased following incubation with the supernatant fluids of pMΦ for 7 days to a similar extent as that of MSCs. c-TnI, one of troponin subunits, regulates Ca^2+^-activated tension in cardiac and striated muscle, but not smooth muscle [24,25,26]. The function of c-TnI is to inhibit tension development in striated muscle until Ca^2+^ binds to another troponin subunit on troponin C (TnC) [24]. c-TnI is considered a unique marker of myocardial tissue [25,26,27,28], and greater differentiation into the cardiomyocyte phenotype is reflected in higher cytoplasmic c-TnI expression. Among the GATA family of zinc finger transcription factors, GATA-4 is expressed in the heart [29]. Knockdown of GATA-4 using an antisense strategy can terminate the differentiation of cardiac progenitor cells (CPC) and induce CPC apoptosis, while its overexpression can lead to ectopic beating cardiomyocytes and even an hypertrophic response [29,30]. Thus, GATA-4 is a mediator of CPC differentiation, plays an important role in progenitor cell proliferation, and is another cardiomyocyte marker. The aforementioned results also indicate that the supernatant fluids of pMΦ induced CSC differentiation into cardiomyocytes and enhanced proliferation of CSCs to a similar extent as MSCs.

In contrast to pro-inflammatory factors, levels of growth factors such as IGF-1, VEGF-α, KGF, and bFGF secreted by the pMΦ group were higher than those of MΦ and LPS-MΦ, and similar to those of MSCs, which is consistent with findings in our previous report [11]. Growth factors are recognized to have anti-apoptotic effects and the capacity to induce cell differentiation [31,32,33]. For example, overexpression of IGF-1 in CSCs enhances the repair of injured cardiac tissue [32] and promotes CSCs differentiation, while VEGF-α replenishes cardiomyocytes by inducing pluripotent stem cell differentiation [33]. Therefore, the induction of CSC differentiation and proliferation by the supernatant fluids of pMΦ may be related to these growth factors. However, the specific mechanisms of this phenomenon were not studied here. Some other key factors, such as miRNA or proteins in the supernatant fluids, need to be found, not just anti-inflammation and growth cytokine. Furthermore, animal experiments and clinical research are warranted to verify the observations and specific mechanisms presented.

The findings of the present study provide new insight into the therapeutic effect of MSCs after transplantation into a myocardial infarction model, and serve as the basis for future research into the specific mechanisms of macrophage phenotype change, CSC differentiation, and the various cytokines secreted by pMΦ. Further animal experiments and clinical research are warranted to verify the observations presented.

## 4. Materials and Methods

### 4.1. Animals

C57BL/6 mice (6 to 10 weeks old) were purchased from Slac Laboratory Animal Co., Ltd. (Shanghai, China) and kept under specific pathogen-free conditions. All animal experiments were approved by the Ethics Committee of Xinhua Hospital affiliated with Shanghai Jiaotong University School of Medicine, Shanghai China and the approval No. was XHEC-F-2016-003.

### 4.2. Cell Isolation and Culture

Seven-to-10 week-old male C57BL/6 mice were used to isolate bone marrow-derived MSCs. The mice were anesthetized by using pentobarbital (50 mg/kg, intraperitoneal injection) to minimize suffering and killed by cervical dislocation without recovery from anesthesia. Tibias and femurs then were removed in sterile conditions. The bone ends were cut and the bone marrow was flushed using PBS (Gibco, Carlsbad, CA, USA, Cat#8115178) supplemented with 1%–2% Fetal Bovine Serum (FBS, Gibco, Cat#10099-141). The cell suspension was then filtered through a 40 µm strainer (BD Falcon, Franklin Lakes, NJ, USA, Cat#352340). After centrifugation of the filtered suspension at 1000 rpm/min for 5 min, the bone marrow cells were cultured in Dulbecco’s Modified Eagle Medium: Nutrient Mixture F-12 (DMEM/F12, HyClone, Beijing, China, Cat**#**SH30023.FS) supplemented with 10% FBS and 1% penicillin–streptomycin (Gibco, Cat#C0222). The culture medium was changed on the fifth day to remove non-adherent cells, and the cells were passaged two days later. MSCs were passaged by using Trypsin (0.25%) including Ethylene Diamine Tetraacetic Acid (EDTA) (Gibco, Cat#25200-056) and identified by flow cytometry (FCM, BD Biosciences, San Jose, CA, USA). The ensuing experiments used passage three cells.

Six-to-8 week-old male C57BL/6 mice were used to isolate peritoneal macrophages. The mice were injected with 1 mL thioglycolate (4%, Sigma-Aldrich, St. Louis, MO, USA, Cat#B2551) and sacrificed 4 days later. After using scissors to cut open the skin over the abdominal area without puncturing the peritoneum, a 10 mL syringe with an 18 G needle was used to inject 10 mL *Roswell* Park Memorial Institute-1640 (RPMI-1640 HyClone, Cat#**SH30255.FS**) with 10% FBS into the abdominal cavity. The belly then was pressed about 20 times, and media was withdrawn using a syringe and subsequently injected into six well plates and incubated at 37 °C for 3–4 h. Supernatant fluid was aspirated and the cells were washed thrice with PBS before being incubated overnight. Cells were collected on the following day and labeled with PE-anti-F4/80 (eBioscience, Carlsbad, CA, USA, Cat#12-4801-80) to identify peritoneal macrophages by FCM.

Cardiac stem cells were obtained from the hearts of 7–8 week-old male C57BL/6 mice via a two-step protocol, as previously described [34,35]. Firstly, the hearts of mice were chopped and cultured for 2–3 weeks until the small, round, phase-bright cells migrated from the adherent heart tissue and proliferated over a fibroblast layer. Then, the small, round, phase-bright cells were isolated through magnetic-activated cell sorting (MACS) with Sca-1 magnetic beads (MiltenyiBiotec Inc., Auburn, CA, USA) to obtain Sca-1 + cells as instructed by the manufacturers’ protocols. The selected Sca-1 cells were cultured and maintained in ISCOVE’S modified DMEM (IMDM, HyClone, Cat#SH30228.FS) supplemented with 20% FBS, 1% penicillin–streptomycin, 1% l-glutamine (Sigma-Aldrich, Cat#59202C) and 0.0068 µL/mL β-mercaptoethanol (Sigma-Aldrich, Cat# 07604).

### 4.3. GFP Transfection

A mouse lentivirus carrying GFP reporter genes (Bio-Link, SHH, Shanghai, China) was transfected into MSCs at passage 4. MSCs were seeded in a six-well plate at 1 × 10^5^ cells per well, grown overnight. Next day, cell culture was aspirated and 1 mL cell medium without penicillin–streptomycin was added into MSCs which contain 5 µg/mL polybrene and lentivirus at a MOI of 1, 5, and 10. After transfection for 12 h, virus-containing supernatant was aspirated and replaced with complete culture medium. Fluorescence microscopy was used to observe transfection efficiency, and transfection rate was evaluated by FCM after 48 h. The optimal MOI was chosen for highest GFP expression.

### 4.4. Detection of MSC Apoptosis and Isolation of pMΦ

The three groups in this experiment were: normal; 0.5% O_2_ for 6 h and cultured with PBS; and 0.5% O_2_ for 24 h and cultured with PBS. Then, dMSCs in these three groups were measured by FCM. Annexin V-PE/7-AAD (eBioscience, Cat#559763) double staining was used to identify the apoptosis rate of MSCs. The cells (2 × 10^6^ cells/mL) were harvested, washed twice with ice-cold PBS, and incubated for 15 min in 1X Annexin V Binding Buffer containing 7-AAD-Percp and Annexin V-PE. Finally, apoptosis was detected by FCM and analyzed by the FlowJo software (Tree Star Inc., Ashland, OR, USA). Experiments were carried out in triplicate.

dMSCs of the third group were plated in six-well plates, and macrophages were immediately added to each well with or without of LPS (50 ng/mL, Sigma-Aldrich, Cat#L2630). Co-cultured cells were incubated for another 48 h, and non-adherent cells (i.e., dMSCs that were not phagocytized by macrophages) were removed. According to FITC-GFP and PE-anti-F4/80, the adherent cells were sorted by FCM to obtain pMΦ, which then were incubated for an additional period of 24 h to obtain the supernatant fluids. Supernatant fluids were collected and stored at −20 °C until use, and cells were collected into a centrifuge tube for real-time PCR. Phagocytosis was evaluated by fluorescence microscope (Leica, Wetzlar, Germany).

### 4.5. Cell Viability Assay

CCK-8 assay was used to measure the viability of CSCs. CSCs (1000/well) were seeded in 96-well plates overnight and then incubated with the supernatant fluids of MΦ, LPS + MΦ, pMΦ, and MSCs. Each group was tested in triplicate in three replicate wells. Ten µL CCK-8 solution (DOJINDO, Kumamoto, Japan, Cat#AY-4710P) was added into the cells of the four groups at 0, 6, 12, and 24 h, respectively. After incubation for another 4 h, the optical density (OD) values were determined at 450 nm by using a microplate reader (BioTek, Winooski, VT, USA) at each time point.

### 4.6. Real-Time PCR Analysis

Expression of IGF-1, VEGF-Α, KGF, bFGF, IL-10, TGF-β, IL-6, and TNF-α in MΦ, LPS + MΦ, pMΦ, and MSC cells was tested by real-time PCR Analysis. Total RNA was obtained from the cells in the four groups using TRIzol reagent (Takara, Dalian, China, Cat#RR036A) following the manufacturer's instructions, and 1 µg of total RNA was reverse-transcribed to cDNA using the PrimeScript RT Master Mix kit (Takara) according to the manufacturer’s instructions. The SYBR Premix Ex TaqTM kit (Takara, Cat# RR420A) in a total volume of 20 with 2 µL of cDNA primers (0.2 mM each), 10 µL of SYBR Green, and 0.4 µL Rox Dye II was used to perform the real-time PCR Analysis. The samples were run on an ABI 7500 detector (Applied Biosystems, Foster City, CA, USA) with standard PCR conditions: 95 °C for 30 s, followed by 40 cycles of 95 °C for 5 s and 60 °C for 34 s, with a final dissociation stage. GAPDH cDNA (Sangon Biotech, Shanghai, China) was used as endogenous control to normalize the *C*t value for target genes. The sequences of the primers (Sangon Biotech) for the target genes are listed in Table 1.

### 4.7. Flow Cytometry (FCM) Analysis

Anti-hypoxic activity and CSC markers were detected by FCM Analysis. CSCs were incubated with the supernatant fluids of MΦ, LPS + MΦ, pMΦ, and MSCs. Cells were then harvested after 0, 6, 12, and 24 h in hypoxia (0.5% O_2_), respectively. Apoptosis of CSCs was determined using the Annexin V-PE/7-ADD as previously described. Three steps were followed to determine CSC markers. In step one, the cells were fixed with 4% paraformaldehyde (Fushen, Shanghai, China, Cat#FS030101) for 10 min and then permeabilized with 0.1% PBS-Tween20 (Beyotime, Shanghai, China, Cat#ST825) for 20 min. In step two, the cells were washed thrice with PBS and resuspended with 100 µL PBS, and then primary antibody was added for 30 min at room temperature. The secondary antibody was added for 30 min at room temperature, and the cells were washed thrice and resuspended with 100 µL PBS. Then, the cells were washed and resuspended in PBS and identified by FCM; the results were analyzed using FlowJo software. The primary antibody against GATA-4 (Cat#ab134057) and TnI (Cat#ab47003) and the secondary antibodyAlexa Fluor^®^ 488 goat anti-rabbit IgG (H + L) (Cat#ab150077) were purchased from Abcam (Cambridge, UK), and used at the recommended concentrations in experiments carried out in triplicate.

### 4.8. Statistical Analysis

Statistical analysis was performed using SPSS software 19.0 (SPSS Inc., Chicago, IL, USA). All values are expressed as mean ± SD. One-way analysis of variance (ANOVA) was used to assess the effects of one factor among multiple groups, and a Tukey test was used for post hoc testing. Statistical significance was defined as *p* < 0.05.

## 5. Conclusions

Activated macrophages that had phagocytized dMSCs (termed pMΦ) expressed higher levels of anti-inflammatory cytokines, lower levels of pro-inflammatory cytokines, and secreted many growth factors. CSCs incubated with the supernatant fluids of said pMΦ exhibited enhanced anti-hypoxic and proliferative activities and differentiation into cardiomyocytes. The latter findings provide us a new understanding about the mechanisms underlying the therapeutic effect of MSCs after transplantation into a myocardial infarction model. Future study should focus on the extraction of some miRNAs or proteins from the supernatant fluids that could directly act as a therapy for myocardial infarction. These results provide us with a new horizon for the treatment of myocardial infarction.

## Figures and Tables

**Table 1:** Sequences of forward and reverse primers for target genes.

Target Gene	Forward and Reverse of Prime
*IL-10*	5’-TGCACTACCAAAGCCACAAG-3’; 5’-TGATCCTCATGCCAGTCAGT-3’
*Il-6*	5’-TCTGCAAGAGACTTCCATCCA-3’; 5’-AGTCTCCTCTCCGGACTTGT-3’
*TNF-α*	5’-GGTGCCTATGTCTCAGCCTC-3’; 5’-CCACTTGGTGGTTTGTGAGTG-3’
*TGF-β*	5’-ATTCCTGGCGTTACCTTGG-3’; 5’-AGCCCTGTATTCCGTCTCCT
*IGF-1*	5’-GGGCTGAGCTGGTGGATG-3’; 5’-CTCCAGTCTCCTCAGATCAC-3’
*PGE2*	5’-GGAGACTCTTCGAGGAGCACTT-3’;5’-GGCGATTTAGCAGCAGATATAAGAA-3’
*KGF*	5’-CTGCCAACTCTGCTCTACAG-3’; 5’-TCCAACTGCCACGGTCCTGAT-3’
*bFGF*	5’-AAGGGAGTGTGTGCCAACC-3’; 5’-GCCCAGTTCGTTTCAGTGC-3’
